# Dynamic B_0_ field shimming for improving pseudo‐continuous arterial spin labeling at 7 T

**DOI:** 10.1002/mrm.30387

**Published:** 2024-12-06

**Authors:** Yang Ji, Joseph G. Woods, Hongwei Li, Thomas W. Okell

**Affiliations:** ^1^ Wellcome Centre for Integrative Neuroimaging, FMRIB Division, Nuffield Department of Clinical Neurosciences University of Oxford Oxford UK; ^2^ Department of Electronic Engineering and Information Science, School of Information Science and Technology University of Science and Technology of China Hefei People's Republic of China; ^3^ Institute of Science and Technology for Brain‐inspired Intelligence Fudan University Shanghai People's Republic of China

**Keywords:** 7 T, B0 inhomogeneity, dynamic B0 field shimming, pseudo‐continuous arterial spin labeling (PCASL)

## Abstract

**Purpose:**

B_0_ field inhomogeneity within the brain‐feeding arteries is a major issue for pseudo‐continuous arterial spin labeling (PCASL) at 7 T because it reduces the labeling efficiency and leads to a loss of perfusion signal. This study aimed to develop a vessel‐specific dynamic B_0_ field shimming method for 7 T PCASL to improve the labeling efficiency by correcting off‐resonance within the arteries in the labeling region.

**Methods:**

We implemented a PCASL sequence with dynamic B_0_ shimming at 7 T that compensates for B_0_ field offsets in the brain‐feeding arteries by updating linear shimming terms and adding a phase increment to the PCASL RF pulses. Rapidly acquired vessel‐specific B_0_ field maps were used to calculate dynamic B_0_ shimming parameters. We evaluated both 2D and 3D variants of our method, comparing their performance against the established global frequency offset and optimal encoding scheme‐based corrections. Cerebral blood flow (CBF) maps were quantified before and after corrections, and CBF values from different methods were compared across the whole brain, white matter, and gray matter regions.

**Results:**

All off‐resonance correction methods significantly recovered perfusion signals across the brain. The proposed vessel‐specific dynamic B_0_ shimming method improved the labeling efficiency while maintaining optimal static shimming in the imaging region. Perfusion‐weighted images demonstrated the superiority of the 3D dynamic B_0_ shimming method compared to global or 2D‐based correction approaches. CBF analysis revealed that 3D dynamic B_0_ shimming significantly increased CBF values relative to the other methods.

**Conclusion:**

Our proposed dynamic B_0_ shimming method offers a significant advancement in PCASL robustness and effectiveness, enabling full utilization of 7 T ASL high sensitivity and spatial resolution.

## 1 INTRODUCTION

Arterial spin labeling (ASL) offers a noninvasive method for quantifying tissue perfusion by leveraging endogenous blood water protons as a contrast agent.^1^, ^2^ Pseudo‐continuous arterial spin labeling (PCASL) stands out among various ASL methods due to its superior labeling efficiency to continuous ASL (CASL), higher SNR than pulsed ASL (PASL), and greater compatibility with modern clinical scanners, making it ideal for clinical MRI applications.^3^, ^4^ ASL typically involves two acquisitions: a labeling scan, where blood water magnetization is inverted; and a control scan, where magnetization remains unaltered. Perfusion information is then extracted by subtracting the labeled scan from the control scan. Whereas this method effectively captures tissue perfusion, the low blood water concentration and T_1_ decay of the labeled blood inherently result in a low SNR. This characteristic presents significant challenges in achieving high‐quality perfusion measurements. Ultrahigh field (UHF) MRI has emerged as a promising avenue to boost ASL SNR due to several factors.^5^ Firstly, the intrinsic SNR exhibits a theoretical dependence on the static magnetic field strength (B_0_) following a power law relationship, scaling with B_0_^1.94 ± 0.16^.^6^ Secondly, the extended ASL tracer lifetime (i.e., blood T_1_) at a higher field indirectly contributes to increased SNR in ASL.^7^, ^8^ However, implementing ASL sequences at UHF presents challenges, primarily due to specific absorption rate (SAR) limitations as well as B_0_ field and transmit B_1_ field (B_1_^+^) inhomogeneities, especially in the neck. These inhomogeneities can substantially reduce the labeling efficiency, thereby reducing the perfusion signal. In severe cases where there are significant offsets from the nominal B_0_ in PCASL, the perfusion signal can shrink to zero, or the labeling and control scans may even be completely reversed. It is crucial to recognize that B_0_ field inhomogeneities vary considerably across different vessels, individuals, and even repeated scans, influenced by factors such as static shimming, subject positioning, placement of the labeling region, and individual anatomical differences.^9^, ^10^ This variability poses a substantial challenge in achieving consistent cerebral blood flow (CBF) measurements with ASL at UHF, thereby impacting its accuracy and reproducibility.

Various compensation methods have been explored to address this challenge. A straightforward approach using static shimming applied to both labeling and imaging regions partially alleviates the issue,^11^ but residual inhomogeneity remains problematic. Additionally, spatial separation between the labeling and imaging regions presents an additional challenge for shimming and can lead to suboptimal shimming settings for both regions, ultimately compromising image quality (e.g., distortion/signal dropout for EPI) and labeling efficiency. Unbalanced PCASL with optimized parameters has been shown to offer improved performance for off‐resonance effects, but significant signal loss persists at larger phase offsets, limiting its effectiveness.^12^ Multi‐phase PCASL (MP‐PCASL) addresses off‐resonance issues by acquiring data at multiple RF phase increments instead of the conventional two (0 and π).^13^ This enables a more precise fit of voxel data to predicted response curves, reducing phase mismatches and resulting in more accurate and reliable CBF measurements. However, MP‐PCASL suffers from lower efficiency or increased scan times, which may limit its widespread adoption in research and clinical settings. A strategy was initially proposed to address B_0_ field inhomogeneity in the labeling region of PCASL scans by applying an additional z‐gradient and RF phase adjustment.^9^ This method uses a coronal field inhomogeneity map to identify field offsets for arteries at various z‐axis locations. Based on these field offsets and corresponding z‐axis location information, a z‐gradient term and an additional RF phase term are calculated to compensate for z‐direction B_0_ field inhomogeneity and residual global frequency offset, respectively. Whereas this approach effectively mitigates B_0_ field inhomogeneity, it does not account for in‐plane variations. This limitation becomes crucial when the labeled feeding arteries have significantly different off‐resonance values. OptPCASL offers a more comprehensive solution by addressing B_0_ field inhomogeneity, including both the global frequency offset and the local variations between the labeled feeding arteries.^14^ This approach accounts for the global phase error, which affects all feeding arteries uniformly and the local phase errors specific to individual feeding arteries. It achieves this by incorporating an additional compensation RF phase term into both the tag and control conditions of the ASL sequence, and by applying extra in‐plane gradient pulses between the RF tagging pulses. A very similar method has been developed at 7 T,^15^ but both methods utilize an MP‐PCASL pre‐scan to measure the phase tracking errors that are used to estimate the RF phase increment and transverse gradients required, which extends the total scan time. An optimized encoding scheme (OES)‐based method has also been proposed to correct for off‐resonance at an arbitrary number of vessel locations using a rapidly acquired field map of the labeling plane.^10^, ^16^ The OES is a Fourier‐based technique that calculates the optimum transverse gradient blips and RF phases to apply to account for phase accrual due to off‐resonance. Whereas the three aforementioned methods effectively address frequency offset arising from in‐plane B_0_ field variations across the vessels, they do not account for through‐plane B_0_ field inhomogeneity. This could be problematic if the field variations of arteries along the through‐plane are significant.

In this work, we propose a novel dynamic B_0_ shimming method to improve PCASL labeling efficiency at 7 T by directly addressing B_0_ field inhomogeneity. Unlike conventional static B_0_ shimming, which maintains constant shimming settings throughout the entire scan, this method dynamically adjusts the linear (gradient) shimming currents between the labeling and non‐labeling periods, providing optimal B_0_ homogeneity for both labeling and imaging. This proposed correction method addresses both in‐plane and through‐plane B_0_ field inhomogeneities within the labeled arteries. By targeting the B_0_ field inhomogeneity specifically within the feeding arteries, rather than the entire labeling region (including static tissue), this method achieves significantly enhanced correction effectiveness. We compared our proposed method to existing off‐resonance correction strategies for 7 T PCASL, demonstrating its effectiveness and superiority. This study highlights the feasibility of our approach, achieving high‐quality PCASL perfusion data in the whole human brain at 7 T. This work builds upon previously presented research in abstract form.^17^

## 2 METHODS

### 2.1 PCASL implementation with dynamic B_0_ shimming

**FIGURE 1:**
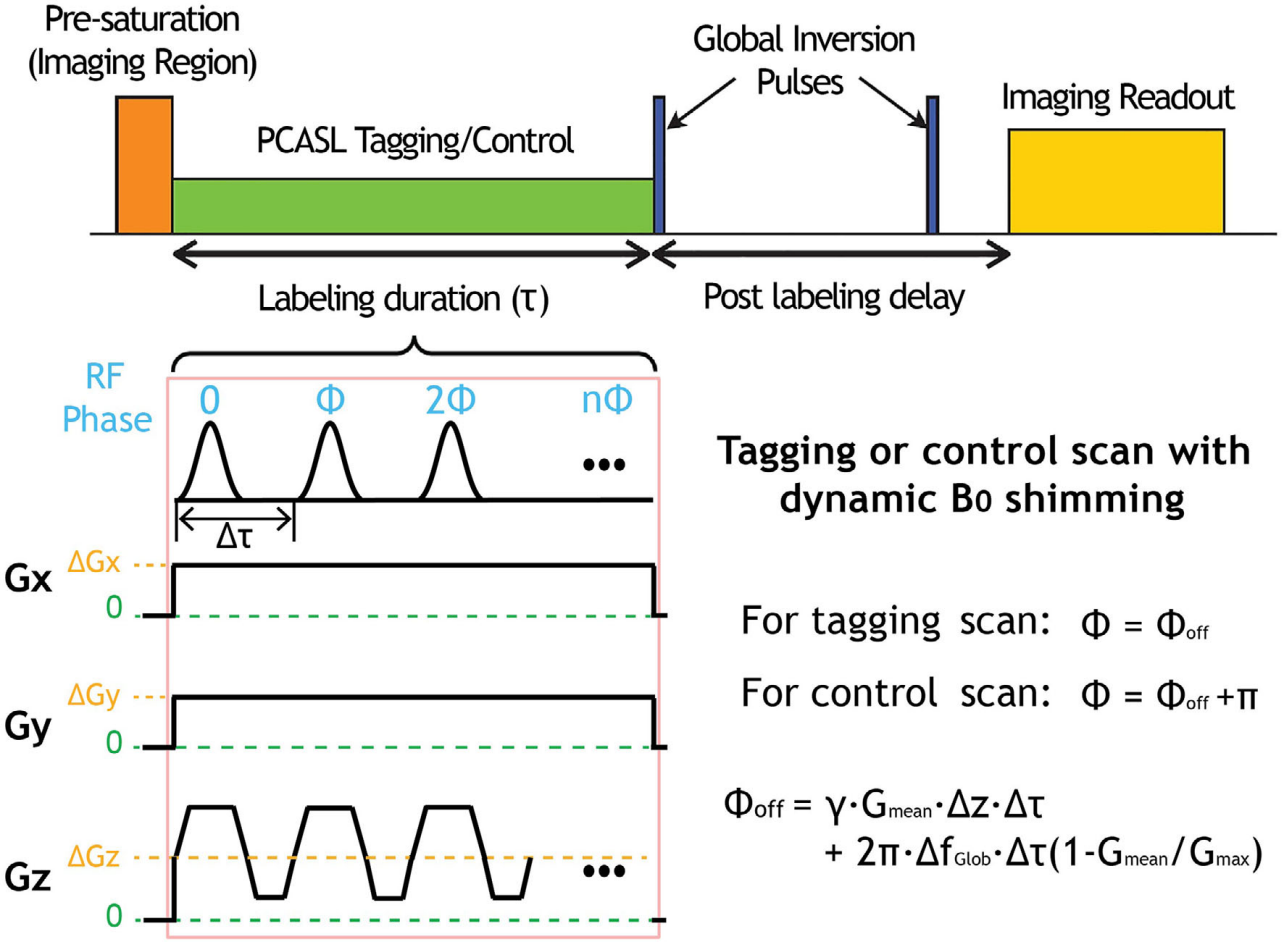
This diagram illustrates the proposed PCASL sequence with integrated dynamic B_0_ shimming for field inhomogeneity correction. The PCASL module consists of a series of discrete slice‐selective RF pulses with interval Δτ. For field inhomogeneity compensation, additional gradients with constant amplitude are applied along three orthogonal axes during the PCASL pulse train and deactivated during the post‐labeling delay and EPI readout phases. The sequence also incorporates specific phase increments into the PCASL RF pulses to correct for a residual global frequency offset ($∆f_{\text{Glob}}$). Note that $\Phi_{\mathrm{off}}$ includes both phase corrections for global off‐resonance and the off‐isocenter of the labeling plane. PCASL, pseudo‐continuous arterial spin labeling.

The proposed PCASL sequence diagram with dynamic B_0_ shimming is illustrated in Figure 1. The PCASL tagging/control module consists of a train of discrete slice‐selective RF pulses applied at intervals of Δτ. To correct B_0_ inhomogeneity within the labeling region, the system dynamically adjusts first‐order shimming parameters during the labeling period and resets them to their original values for optimal background suppression and imaging during the post‐labeling delay and EPI readout. Because the first‐order shimming relies on modifying the linear x, y, and z gradients, these adjustments introduce offsets to the gradient amplitudes (i.e., $∆G_{x}$, $∆G_{y}$, $∆G_{z}$) compared to the initial values throughout the PCASL pulse train. The residual global frequency offset ($∆f_{\text{Glob}}$) is corrected by adding additional phase increments to the PCASL RF pulses. Thus, in addition to 0 and π phase, an additional $\Phi_{\mathrm{off}}$ that includes both phase corrections for global off‐resonance and the off‐isocenter of the labeling plane is added into the RF phases for both control and tagging, which is given by^18^:

(1) $$\begin{array}{ll} \Phi_{\mathrm{off}} & =\gamma \cdot G_{\text{mean}}\cdot ∆z\cdot \Delta \tau +2\pi \cdot ∆f_{\text{Glob}}\\ & \;\cdot \Delta \tau \left(1-G_{\text{mean}}/G_{\max}\right), \end{array}$$

where $G_{\text{mean}}$ is the mean gradient, $G_{\max}$ is the maximum gradient, ∆z is the offset of the labeling plane, $∆f_{\text{Glob}}$ is the residual global frequency offset. The calculation of $∆f_{\text{Glob}}$, along with the parameters $∆G_{x}$, $∆G_{y}$ and $∆G_{z}$, are detailed in the following subsection. Note that the (1 – G_mean_/G_max_) correction term accounts for the small shift in the labeling plane location due to the global frequency offset.

### 2.2 Vessel‐specific dynamic B_0_ shimming

The required gradient offset amplitudes ($∆G_{x}$, $∆G_{y}$, $∆G_{z}$) and the residual global frequency offset ($∆f_{\text{Glob}}$) for dynamic B_0_ shimming can be estimated from the field offset information of the vessels within the labeling region. This estimation is achieved by solving the following equation:

(2) $$\frac{\gamma }{2\pi }\left[\begin{array}{l} \begin{array}{llll} {\mathrm{PX}}_{1} & {\mathrm{PY}}_{1} & {\mathrm{PZ}}_{1} & -1\\ {\mathrm{PX}}_{2} & {\mathrm{PY}}_{2} & {\mathrm{PZ}}_{2} & -1\\ {\mathrm{PX}}_{3} & {\mathrm{PY}}_{3} & {\mathrm{PZ}}_{3} & -1 \end{array}\\ \vdots \\ {\mathrm{PX}}_{n}\;{\mathrm{PY}}_{n}\;{\mathrm{PZ}}_{n}\;-1 \end{array}\right]\left[\begin{array}{l} ∆G_{x}\\ ∆G_{y}\\ ∆G_{z}\\ \;2\pi ∆f_{\text{Glob}}/\gamma \end{array}\;\right]=\left[\begin{array}{l} -\;∆f_{1}\\ -\;∆f_{2}\\ -\;∆f_{3}\\ \vdots \\ -\;∆f_{n} \end{array}\right],$$

where [${\mathrm{PX}}_{i}$, ${\mathrm{PY}}_{i}$, ${\mathrm{PZ}}_{i}$], and $∆f_{i}$ represent the location of the i‐th vessel voxel within the 3D labeling region and the corresponding global frequency offset, respectively. Because the labeling efficiency is only affected by the B_0_ homogeneity of the flowing blood inside the brain‐feeding arteries within the labeling region, we restrict the shimming optimization area to small regions of interest (ROIs) encompassing only the relevant voxels. This targeted method allows for much more efficient B_0_ shimming by focusing solely on the relevant vessel voxels within the labeling region, excluding static tissues and air that do not impact the labeling process. Additionally, dynamic B_0_ shimming can also be performed in 2D when ignoring through‐plane B_0_ variations:

(3) $$\frac{\gamma }{2\pi }\left[\begin{array}{l} \begin{array}{lll} {\mathrm{PX}}_{1} & {\mathrm{PY}}_{1} & -1\\ {\mathrm{PX}}_{2} & {\mathrm{PY}}_{2} & -1\\ {\mathrm{PX}}_{3} & {\mathrm{PY}}_{3} & -1 \end{array}\\ \vdots \\ \begin{array}{lll} {\mathrm{PX}}_{n} & {\mathrm{PY}}_{n} & -1 \end{array} \end{array}\right]\left[\begin{array}{l} ∆G_{x}\\ ∆G_{y}\\ \;2\pi ∆f_{\text{Glob}}/\gamma \end{array}\right]=\left[\begin{array}{l} -\;∆f_{1}\\ -\;∆f_{2}\\ -\;∆f_{3}\\ \vdots \\ -\;∆f_{n} \end{array}\right],$$

Under such conditions, the shimming gradient's offset along the z‐direction should be set to zero. Similarly, if the through‐plane B_0_ variations are only considered and the offset amplitudes for the transverse plane gradients (x and y) are instead set to zero, this method essentially reduces to the one proposed by Jahanian et al.,^9^ as mentioned above.

**FIGURE 2:**
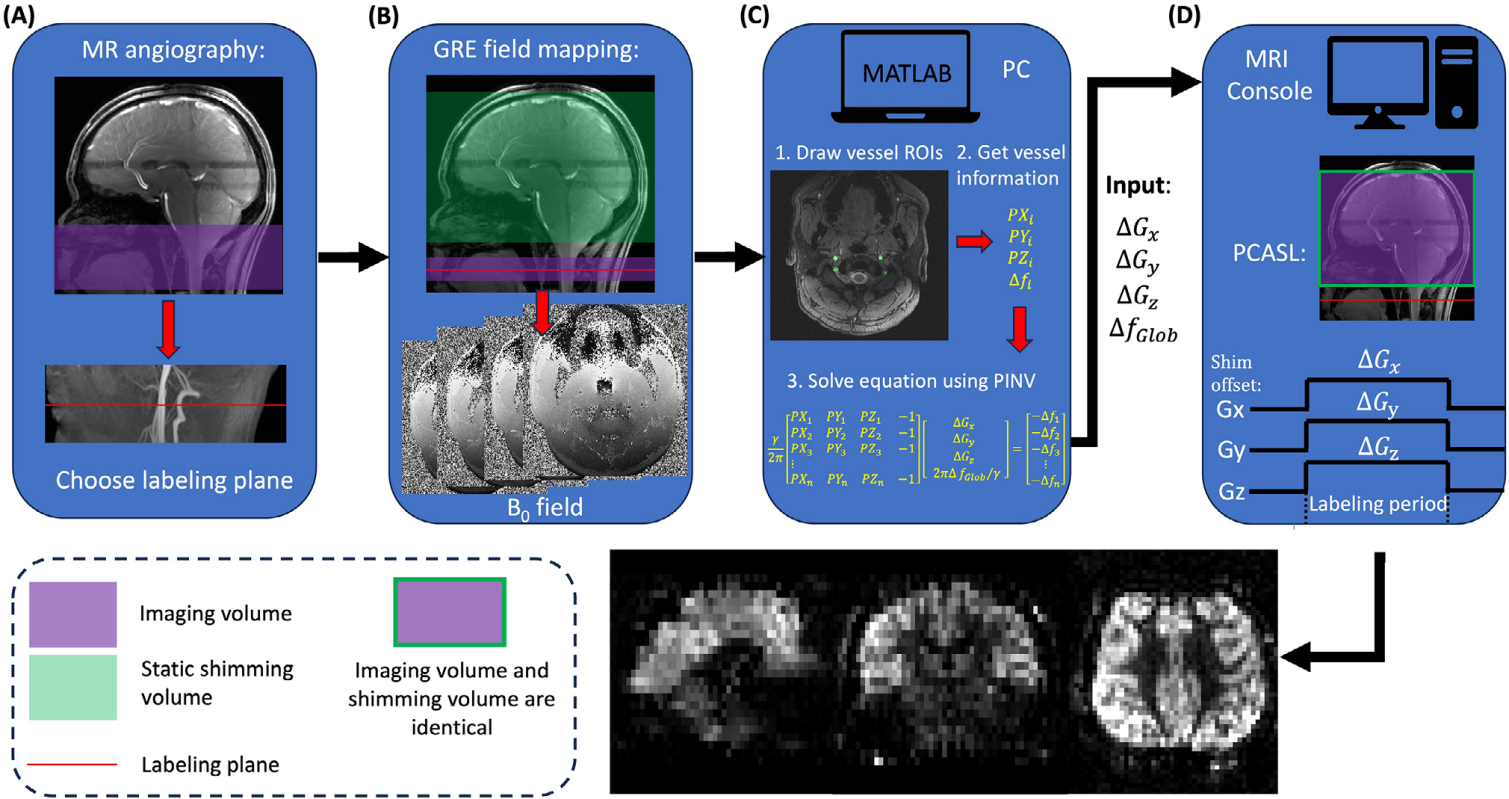
Workflow for using dynamic B_0_ shimming in PCASL. (A) Step 1: Perform a TOF sequence to identify the optimal labeling plane, positioning it at the middle of the V3 segment of the vertebral arteries. (B) Step 2: Conduct a 2D multi‐slice GRE field mapping sequence centered at the labeling plane, covering the labeling region, to map the field offset of the vessel voxels within this area. Note that the field map's FOV is assumed to cover the region where blood labeling occurs. (C) Step 3: Manually draw the vessel ROIs within the labeling region using MATLAB R2023b (MathWorks, Natick, MA). Extract the field offset and corresponding coordinates of each voxel to formulate the equation, then use MATLAB's PINV function to calculate the required gradient offsets and global frequency offset. (D) Step 4: Input the calculated values into the MRI console and run the PCASL with the static B_0_ shimming volume matching that of the GRE field mapping sequence, ultimately generating high‐quality perfusion images. GRE, gradient echo; ROI, region of interest; TOF, time of flight.

Figure 2 illustrates the workflow of 7 T PCASL with dynamic B_0_ shimming. A 3D time‐of‐flight scan is conducted to select the PCASL labeling plane location, which is positioned at the middle of the third segment (V3) of the vertebral arteries,^19^ allowing whole‐brain perfusion measurements. A field mapping sequence is then performed to measure the B_0_ field offset of the labeling region, with its imaging volume centered at the labeling plane. It is important to note that the static B_0_ shimming volume of the field mapping sequence should match that of the subsequent PCASL scan. Vessel ROIs are manually delineated to enable the extraction of field offsets and voxel coordinates from the field map data, facilitating the calculation of dynamic B_0_ shimming‐related parameters. These parameters are then applied during the PCASL scan, which utilizes an identical static B_0_ shimming configuration as the field mapping sequence.

### 2.3 In vivo data acquisition

Data were acquired on a Magnetom 7 T Plus scanner (Siemens Healthineers, Erlangen, Germany) equipped with an 8‐transmit/32‐receive head coil. Six healthy male volunteers (aged 23–34) were scanned under a technical development protocol with approval from local ethical and institutional review boards. A 3D multi‐slab time‐of‐flight angiography sequence with a voxel size of 0.8 × 0.8 × 1.3 mm^3^ was conducted in the neck, enabling the selection of an optimal labeling plane. A rapid 2D multi‐slice dual‐echo gradient echo (GRE) field‐mapping sequence was performed to collect the field offset information of the vessel voxels within the labeling region. Flow compensation gradients were applied along the frequency encoding and slice directions to minimize flow‐induced phase shifts. The main sequence parameters were as follows: TE1 = 4.08 ms, TE2 = 5.1 ms; number of slices = 5; FOV = 220 × 220 mm^2^, TR = 40 ms; in plane spatial resolution = 0.7 × 0.7 mm^2^; slice thickness = 2 mm, scan time = 26 s. To calibrate the transmit voltage for the brain, a 3DREAM B_1_^+^‐mapping sequence^20^ was performed with the imaging volume including the whole brain and the neck. The average voltage from the central slice of the brain was then updated in the system. To mitigate the B_1_^+^ drop‐off at the labeling plane, the nominal flip angle of the B_1_^+^ pulse for labeling was adjusted by multiplying the target flip angle by a factor of V_brain_/V_vessel_, where V_brain_ represents the average voltage across the central slice of the brain, and V_vessel_ represents the average voltage across the labeled vessel voxels.

A previous study has demonstrated that off‐resonance compensation strategies can substantially improve unbalanced PCASL performance at 7 T.^21^ Whereas unbalanced PCASL is less susceptible to off‐resonance effects, it introduces mismatched eddy currents between the label and control conditions, potentially leading to artifacts. Moreover, it is incompatible with vessel‐encoded labeling, a technique we plan to employ in future research. Consequently, we chose balanced PCASL for this study. To evaluate the performance of the proposed dynamic B_0_ shimming method for correcting B_0_ inhomogeneity in PCASL, whole‐brain perfusion images using balanced PCASL with existing correction methods (global frequency offset correction,^21^ OES‐based^10^) and without correction were acquired at 7 T. A PCASL sequence with a 2D GRE EPI readout was employed, utilizing a low‐SAR optimization.^22^ The labeling parameters were as follows: labeling RF pulse = variable‐rate selective excitation Hann‐shaped pulse, RF duration = 530 μs, RF separation = 1060 μs, labeling duration = 1400 ms, target RF flip angle = 9.8° (with the nominal flip angle value on the console adjusted to compensate for the B_1_^+^ drop‐off in the neck, as described above), mean gradient (G_mean_) = 0.20 mT/m, maximum gradient amplitude (G_max_) = 5.5 mT/m, post‐labeling delay = 1800 ms. The aim of incorporating variable‐rate selective excitation was to further reduce SAR while maintaining labeling efficiency.^23^, ^24^ The background suppression pulses we used were nonselective hyperbolic secant inversion pulses. The pulse parameters were: B_1_^+^max = 20 μT, duration = 10.24 ms, β = 763.99 rad/s (giving an amplitude truncation of 4%), and μ = 7.06. The μ value was optimized to maximize inversion efficiency over a B_1_^+^ variation of ±50% and B_0_ variation of ±500 Hz. The pulses were timed to null spins with T_1_ = 1000 and 2000 ms, 100 ms before the first readout using the formula in Günther et al.^25^ The EPI readout employed the following parameters: FOV = 220 × 220 mm^2^, voxel size = 3.4 × 3.4 × 5 mm^3^, matrix size = 64 × 64, flip angle = 90°, phase partial Fourier factor = 6/8, no in‐plane acceleration, TE/TR = 13.0/5200 ms, number of slices = 24, and bandwidth = 2004 Hz/pixel. The minimum TR for our protocol was 4300 ms, but we always added 900 ms of dead time to each TR. This adjustment ensured that we stayed within first‐level SAR limits for all subjects. For the conventional tag/control mode, a total of 41 images, including the M_0_ image and alternating control and tagging images, were acquired, with a total acquisition time of 3:35 min. Additionally, high‐resolution PCASL images were acquired across 24 axial slices, with a voxel size of 2.0 × 2.0 × 4.0 mm^3^, TE/TR = 13.0/6300 ms, and an in‐plane GRAPPA^26^ acceleration factor of 2. Twenty tag‐control image pairs were collected in 4:32 min, without M_0_ acquisition. To enable distortion correction of the EPI images before analysis, field maps covering the perfusion imaging volume were acquired with a static shimming setting matching that of the EPI readout (voxel size: 2.0 × 2.0 × 2.0 mm^3^, TR: 620 ms, ΔTE: 1.02 ms, acquisition time: 2:02 min). Additionally, T_1_‐weighted structural images using MPRAGE were acquired with a voxel size of 0.7 × 0.7 × 0.8 mm^3^ to serve as an anatomical reference.

### 2.4 In vivo data processing

In vivo data were analyzed with the combined use of MATLAB R2023b (MathWorks, Natick, MA) and FMRIB Software Library (FSL) (Oxford, UK).^27^ The vessel ROIs were manually drawn on the magnitude image from the field mapping sequence. The field offset of the vessel voxels was calculated by $∆f=\frac{∆\phi }{2\pi \cdot ∆\mathrm{TE}}$, where ∆TE is the TE difference and ∆ϕ is the phase difference between two echoes. ∆ϕ was computed by taking the angle of a magnitude‐weighted complex sum across channels: $∆\phi =∠\left(\sum_{i}S_{1i}\cdot S_{2i}^{*}\right)$, where $S_{1i}$ and $S_{2i}$ are the complex signals from channel *i* for the first and second echo, respectively, and * denotes the complex conjugate operator. Given a ∆TE of 1.02 ms, which resulted in an approximate dynamic range of ±490 Hz, and considering that the field offset of the vessel voxels was within the range of approximately ±200 Hz, phase unwrapping was not applied during the calculation of the field map for the vessel voxels. However, in more extreme cases, if the B_0_ offset is close to the boundary then phase unwrapping may still be necessary.

Preprocessing of T_1_‐weighted images from MPRAGE was performed using FSL's fsl_anat tool, which included bias correction, brain extraction, and tissue‐type segmentation. Whole‐brain B_0_ field maps for distortion correction were generated using FSL's fsl_prepare_fieldmap tool. All PCASL raw images were motion‐corrected using FSL's mcflirt^28^ tool, with the first (calibration) volume image as a reference. Perfusion‐weighted images were generated by computing the difference between the tag and corresponding control images, and then averaging this difference across all repetitions. The software OXASL^29^, ^30^ (https://github.com/physimals/oxasl, version 0.2.2), which performs Bayesian analysis of ASL MRI data, was used for further CBF quantification. Field maps, preprocessed T_1_‐weighted images, and motion‐corrected PCASL images were provided as input to OXASL. ROIs for white matter (WM) and gray matter (GM) in structural space were generated using FSL_anat. OXASL subsequently registered these ROIs into ASL space, resulting in the ROIs in ASL space.

### 2.5 Bloch simulation and statistical analysis

We conducted a Bloch simulation to determine the thickness of the labeling region in PCASL using low SAR parameters for this study. The simulation was performed using MATLAB R2023b (MathWorks) code, which is available at https://github.com/tomokell/bloch_sim,^31^ with parameters matching those used for in vivo acquisitions. To simplify the interpretation of the labeling region, we assumed T_1_ = T_2_ = ∞ for this simulation. Regional CBF values were calculated within the defined ROIs. Paired *t*‐tests were performed to compare quantitative CBF values obtained from PCASL scans with various correction methods. A value of *p* < 0.05 was considered statistically significant. All data were reported as mean ± SD.

## 3 RESULTS

**FIGURE 3:**
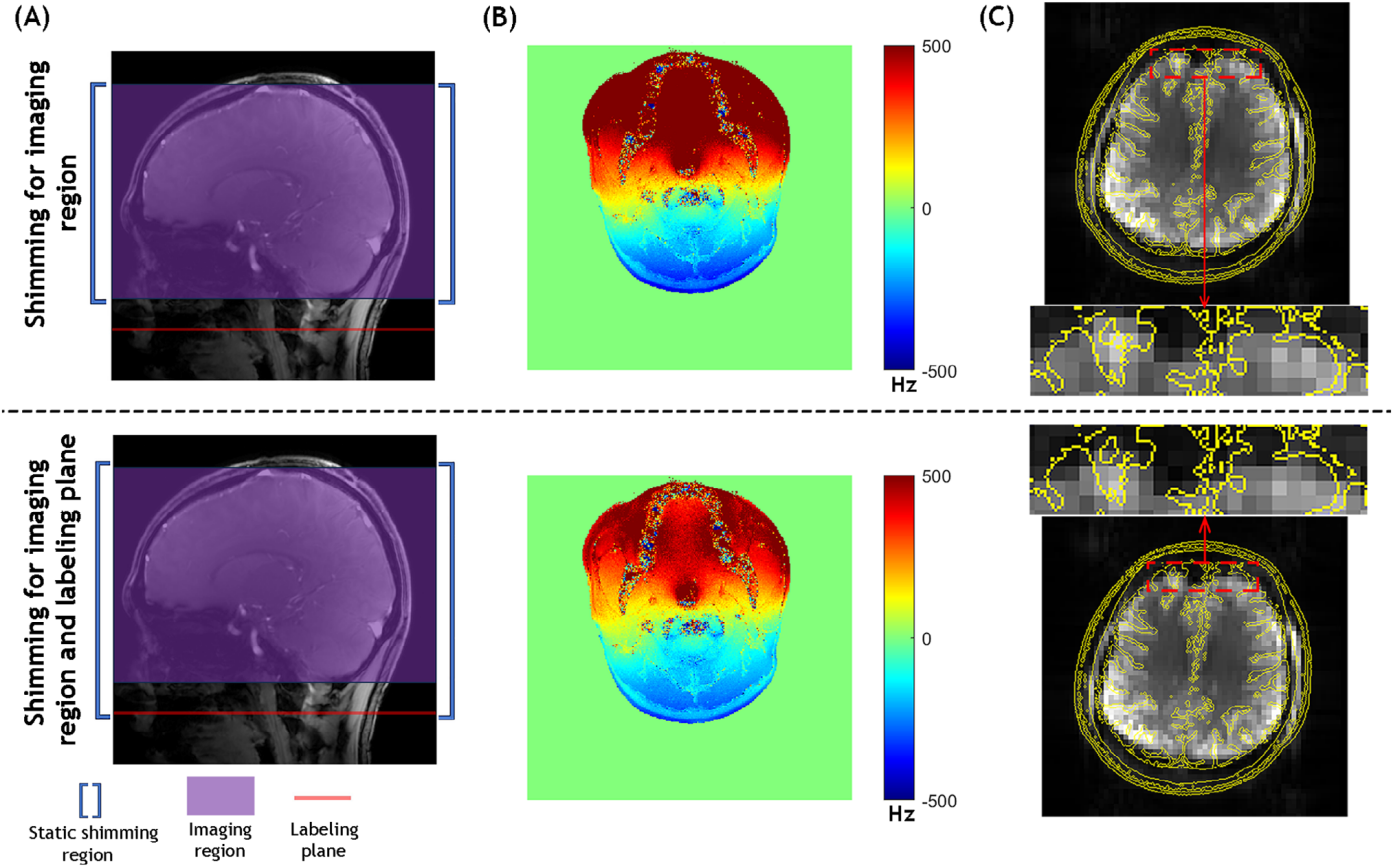
Comparison of two static B_0_ shimming strategies for PCASL. (A) Spatial coverage of static shimming regions illustrated for two approaches: Imaging volume only (upper panel) and combined imaging volume plus labeling plane (lower panel). (B) Representative B_0_ field maps acquired at the labeling plane under both shimming conditions. Note that substantial B_0_ inhomogeneity remains within the labeling plane even when this plane is included in the shimming optimization. (C) Assessment of EPI geometric distortion using MPRAGE structural reference (yellow contour) overlaid on PCASL‐EPI brain images (grayscale). The comparison demonstrates reduced geometric distortion and improved B_0_ homogeneity when shimming is optimized for the imaging volume alone rather than including the labeling plane.

To compare the effects of two different static B_0_ shimming settings on field homogeneity during the labeling and imaging periods of PCASL, field mapping scans at the labeling plane and whole brain PCASL scans were performed on the healthy subjects. Figure 3A illustrates two different static B_0_ shimming setups, one with the shimming optimized only for the imaging volume, whereas the other was optimized for the entire imaging volume with an extended bottom edge to include the labeling plane. Field maps of the labeling plane (Figure 3B) showed a slightly larger field offset when shimming was restricted to the imaging volume compared to the combined imaging and labeling plane shimming. This indicates that static B_0_ shimming that covers both regions is better for the labeling process of PCASL, although considerable off‐resonance remains. Figure 3C shows a representative slice acquired using PCASL‐EPI with the two different static shimming settings, superimposed with the outline of a structural image obtained with MPRAGE. The EPI images from the shimming setting that covers only the imaging region show less distortion, indicating the typical shimming approach for PCASL at 7 T that covers both the imaging region and labeling plane is suboptimal for the imaging readout and background suppression. Consequently, the shimming setup that covers only the imaging volume was used in subsequent PCASL experiments with the dynamic B_0_ shimming correction method.

**FIGURE 4:**
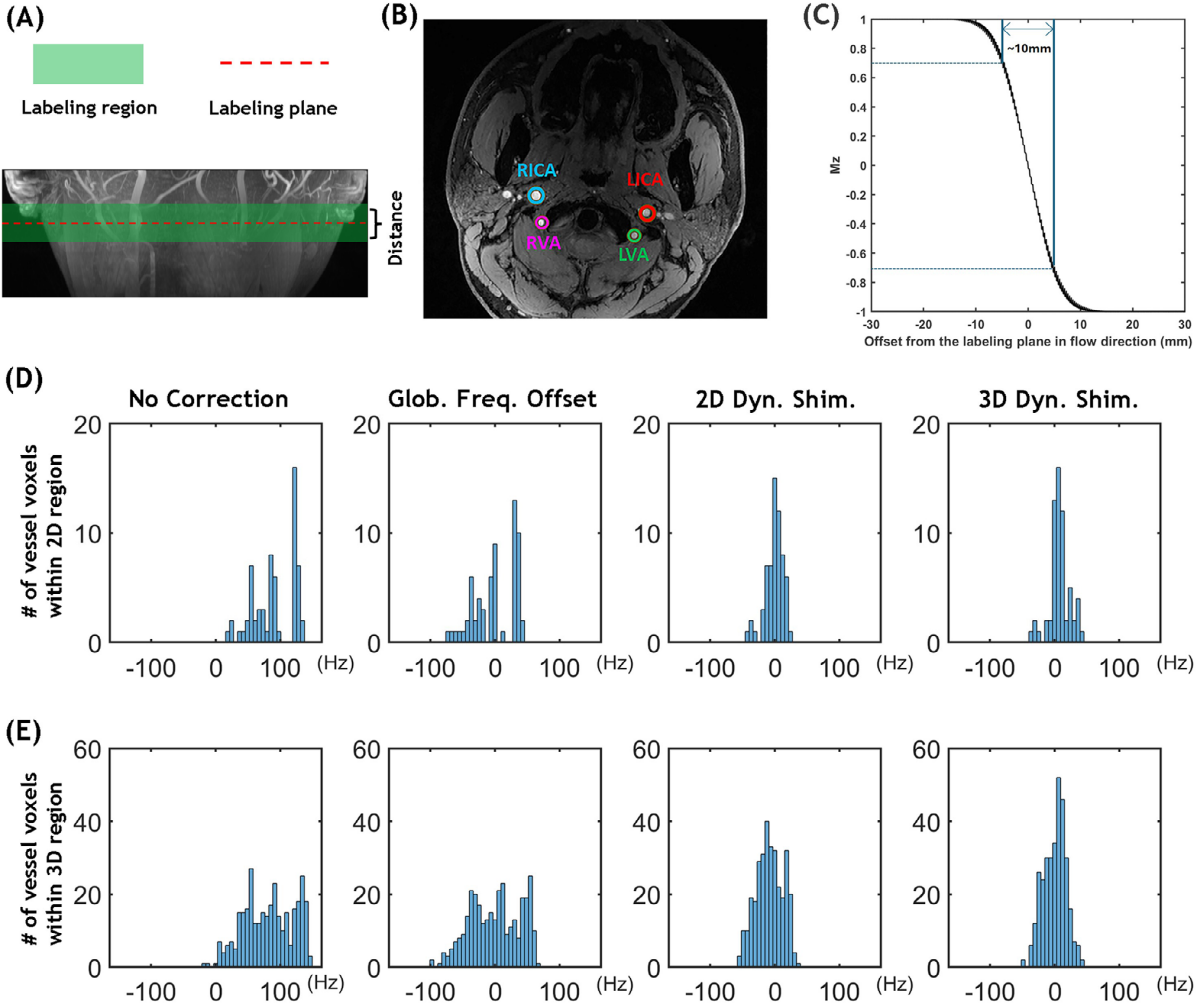
(A) Coronal TOF MIP showing the labeling region (green) where flow‐driven pseudo‐adiabatic inversion occurs in PCASL. The central labeling plane (red dashed line) bisects this region. (B) Axial TOF image at the labeling plane depicting the four major brain‐feeding arteries: RICA, LICA, RVA, and LVA marked with colored circles. (C) Simulation of Mz evolution during PCASL labeling. The labeling region boundaries (approximately 10 mm thickness) were defined using a ± 70% threshold criterion. For simulation clarity, T_1_ and T_2_ relaxation effects were excluded. (D, E) Off‐resonance frequency distributions in vessel voxels from a representative subject, comparing three B_0_ field conditions: Baseline, after global frequency offset correction, and following 2D and 3D dynamic B_0_ shimming, (D) Frequency distributions measured at the single labeling plane slice (2D region). (E) Frequency distributions across the extended labeling region (five consecutive slices centered at the labeling plane, 3D region), demonstrating better B_0_ homogeneity achieved with 3D dynamic shimming. LICA, left internal carotid arteries; LVA, left vertebral arteries; MIP, maximum intensity projection; Mz, longitudinal magnetization; RICA, right internal carotid arteries; RVA, right vertebral arteries.

In PCASL literature, *labeling plane* is often used interchangeably with *labeling region*, even though the labeling region encompasses a larger volume around the plane where the blood is labeled. To avoid confusion in this study, we strictly define the labeling region as the area where blood undergoes longitudinal magnetization inversion, transitioning from a positive threshold value to a negative threshold value; and the labeling plane as the center of the defined labeling region. Note that whereas the labeling plane has a precise position, the boundaries of the labeling region are not explicitly defined in existing literature. Additionally, the thickness of the labeling region is influenced by the specific labeling parameters used. Figure 4A illustrates the spatial relationship between the labeling region and the labeling plane of PCASL, along with the four main brain‐feeding arteries labeled within the labeling plane (Figure 4B). Figure 4C depicts the simulated evolution of the longitudinal magnetization during the PCASL labeling process using our low SAR parameters. In this study, ±70% thresholds of equilibrium magnetization were used to define the labeling region's boundaries, which resulted in a labeling region approximately 10 mm thick. 2D and 3D dynamic B_0_ shimming ROIs were chosen on the vessels within the 2D region (i.e., one slice with 2‐mm slice thickness) and 3D region (i.e., five slices with 2‐mm slice thickness each) centered at the labeling plane, respectively. The average number of vessel voxels within the ROIs for each slice is approximately 80 ± 5. Figure 4D,E show histograms depicting the field offset distribution of the target vessel voxels within the 2D and 3D regions, respectively, before and after correction using global frequency offset, 2D, and 3D dynamic B_0_ shimming methods. Note that the corrected field offset values were based on simulated data, not actual measurements. The histograms clearly demonstrate large field offsets before correction. Whereas all methods successfully reduced off‐resonance, 3D dynamic B_0_ shimming exhibited a narrower distribution of field offsets centered around 0 Hz (the target field offset) in the histogram, indicating improved field homogeneity and more effective shimming.

**FIGURE 5:**
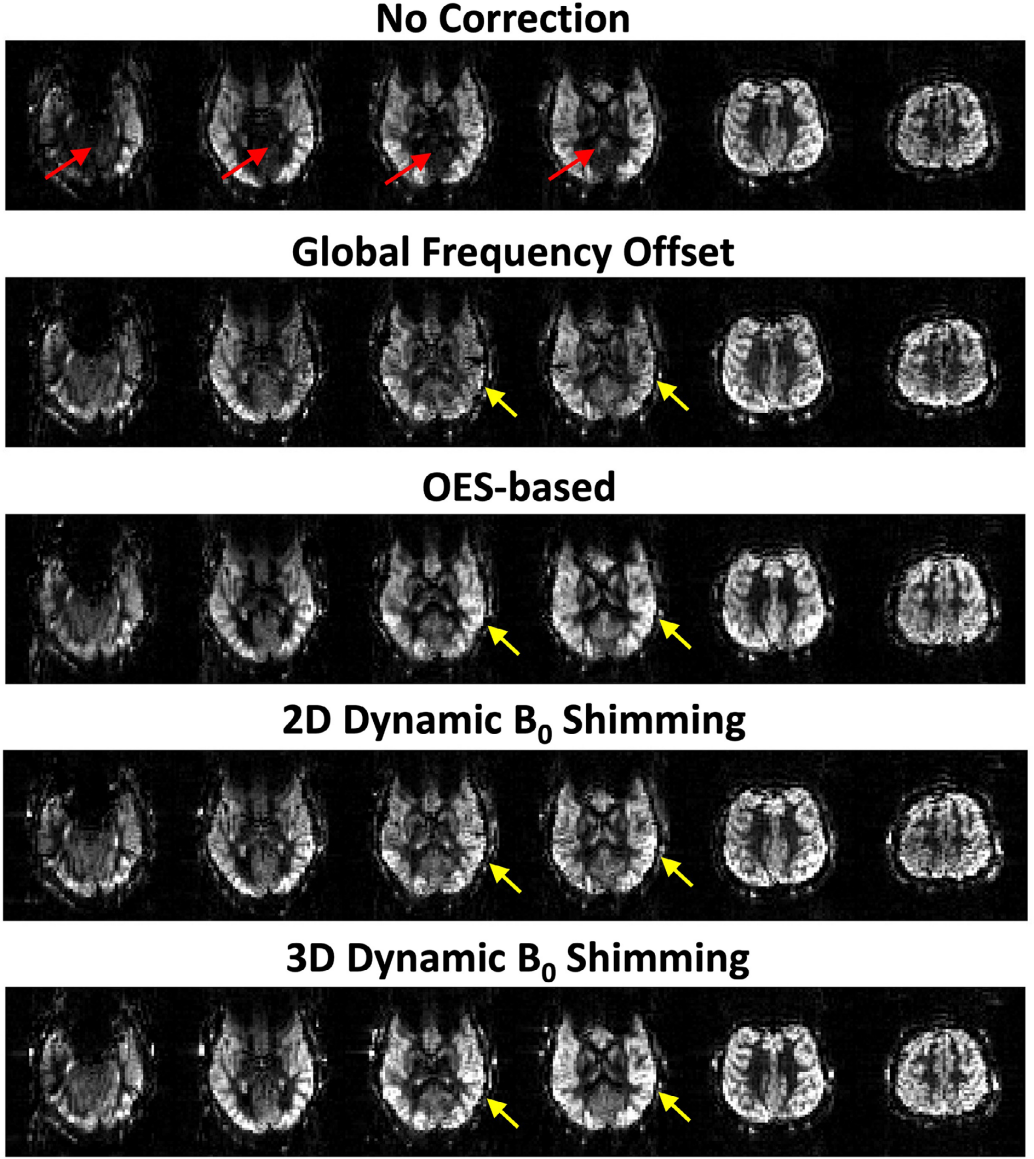
Comparison of whole‐brain perfusion‐weighted images acquired using PCASL with different off‐resonance correction strategies: Uncorrected baseline, global frequency offset correction, OES‐based correction, and 2D/3D dynamic B_0_ shimming. Severe perfusion signal dropout (red arrows) is observed in the posterior cerebral circulation territories in the uncorrected images. All correction methods improve perfusion signal recovery, with varying degrees of effectiveness (yellow arrows highlight regional differences), giving high‐quality perfusion images despite the short scan time (3.5 min). OES, optimized encoding scheme.

Figure 5 compares perfusion‐weighted images from a volunteer obtained using PCASL without and with different off‐resonance correction methods. The images were pair‐wise subtracted and averaged following rigid registration, with no further masking or postprocessing applied. In the uncorrected acquisition, significant perfusion signal loss (indicated by red arrows) is evident in the posterior cerebral circulation due to off‐resonance of the feeding arteries in the labeling region. The signal loss was substantially recovered in all scans after applying various correction methods: global frequency offset, OES‐based, and both 2D and 3D dynamic B_0_ shimming for off‐resonance correction. However, certain brain regions still exhibit lower signal intensity with the global frequency offset method compared to other correction methods (highlighted by yellow arrows). Notably, OES‐based and 2D dynamic B_0_ shimming yielded comparable signal intensity, whereas 3D dynamic B_0_ shimming achieved the highest signal intensity across all regions in this subject.

**FIGURE 6:**
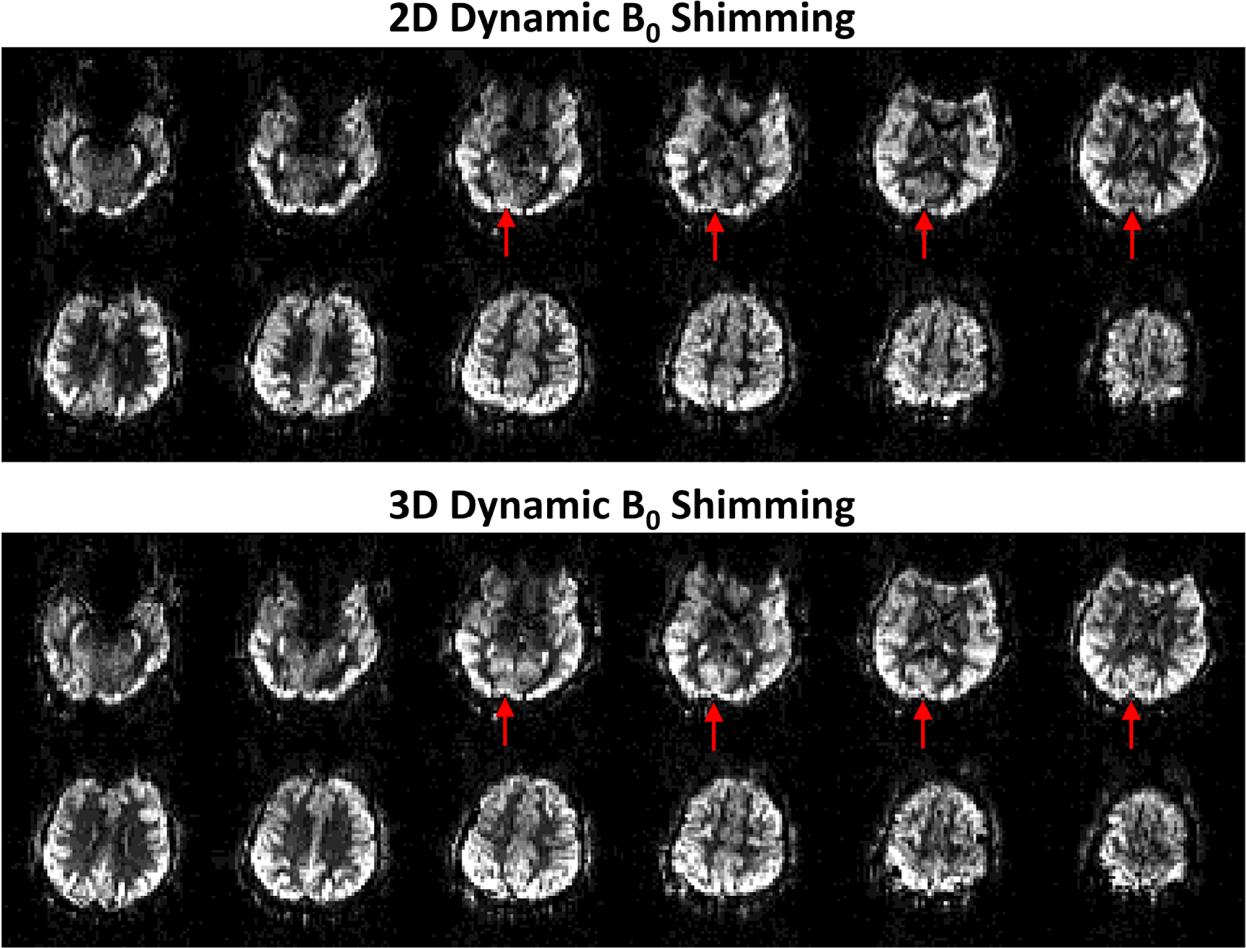
Comparison of whole‐brain perfusion‐weighted images obtained from the same subject (previously shown in Figure 5) using PCASL with 2D and 3D dynamic B_0_ shimming approaches in a different scan. The 3D dynamic B_0_ shimming technique yielded improved perfusion signal in the posterior circulation territories (red arrows), demonstrating its superior performance over the 2D approach.

To highlight the improved performance of the 3D dynamic B_0_ shimming method over the 2D method, Figure 6 compared perfusion‐weighted images using PCASL with both methods in a follow‐up scan of the same subject presented in Figure 5. Both methods produced high‐quality whole‐brain perfusion‐weighted images. However, the 3D shimming method yielded an improved perfusion signal in the posterior circulation, as indicated by the red arrows. It can be inferred that the vertebral arteries exhibit better field homogeneity after the 3D dynamic B_0_ shimming compared to the 2D method.

**FIGURE 7:**
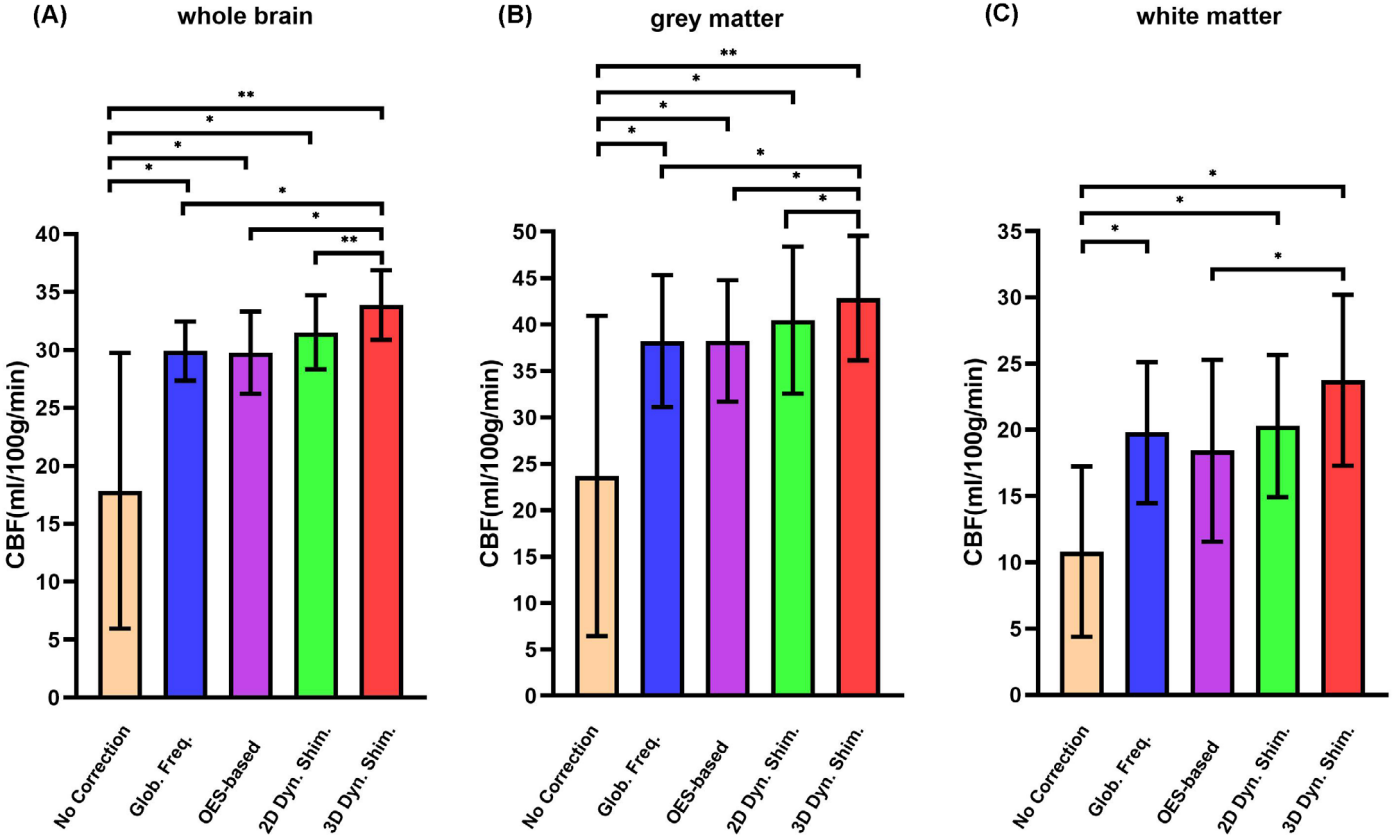
Quantitative comparison of CBF values across different off‐resonance correction methods. Region‐specific CBF measurements in six healthy volunteers comparing baseline (no correction) with four B_0_ correction approaches: Global frequency offset, OES‐based, 2D dynamic B_0_ shimming, and 3D dynamic B_0_ shimming. Analysis is shown for: (A) whole brain, (B) GM, and (C) WM regions. (*p*‐values are indicated by stars: *p < 0.05, **p < 0.001). CBF, cerebral blood flow.

Figure 7 compares the quantitative CBF values within the whole brain, WM, and GM regions across different correction methods in six volunteers. The results indicate that each method produces significantly improved perfusion values for the different brain regions. For the whole brain, the 3D dynamic B_0_ shimming method yields the highest perfusion value of 33.9 ± 3 mL/100 g/min, followed by the 2D dynamic B_0_ shimming and global offset methods, providing 31.5 ± 3.2 and 29.9 ± 2.6 mL/100 g/min, respectively. The OES‐based method provides the lowest value of 29.8 ± 3.5 mL/100 g/min. In GM, the 3D and 2D dynamic B_0_ shimming methods show the highest and second highest values of 42.9 ± 6.7 and 40.5 ± 7.9 mL/100 g/min, respectively, whereas the global offset and OES‐based methods yield similar but lower values of 38.2 ± 7.1 and 38.2 ± 6.5 mL/100 g/min, respectively. In WM, all four methods show a consistent pattern of 23.7 ± 6.4 mL/100 g/min, 20.3 ± 5.4 mL/100 g/min, 19.8 ± 5.3 mL/100 g/min, and 18.4 ± 6.8 mL/100 g/min. This consistent pattern suggests that the 3D dynamic B_0_ shimming method provides the best off‐resonance correction and highest labeling efficiency, which results in higher perfusion values compared to the global offset, OES‐based, and 2D dynamic B_0_ shimming methods.

**FIGURE 8:**
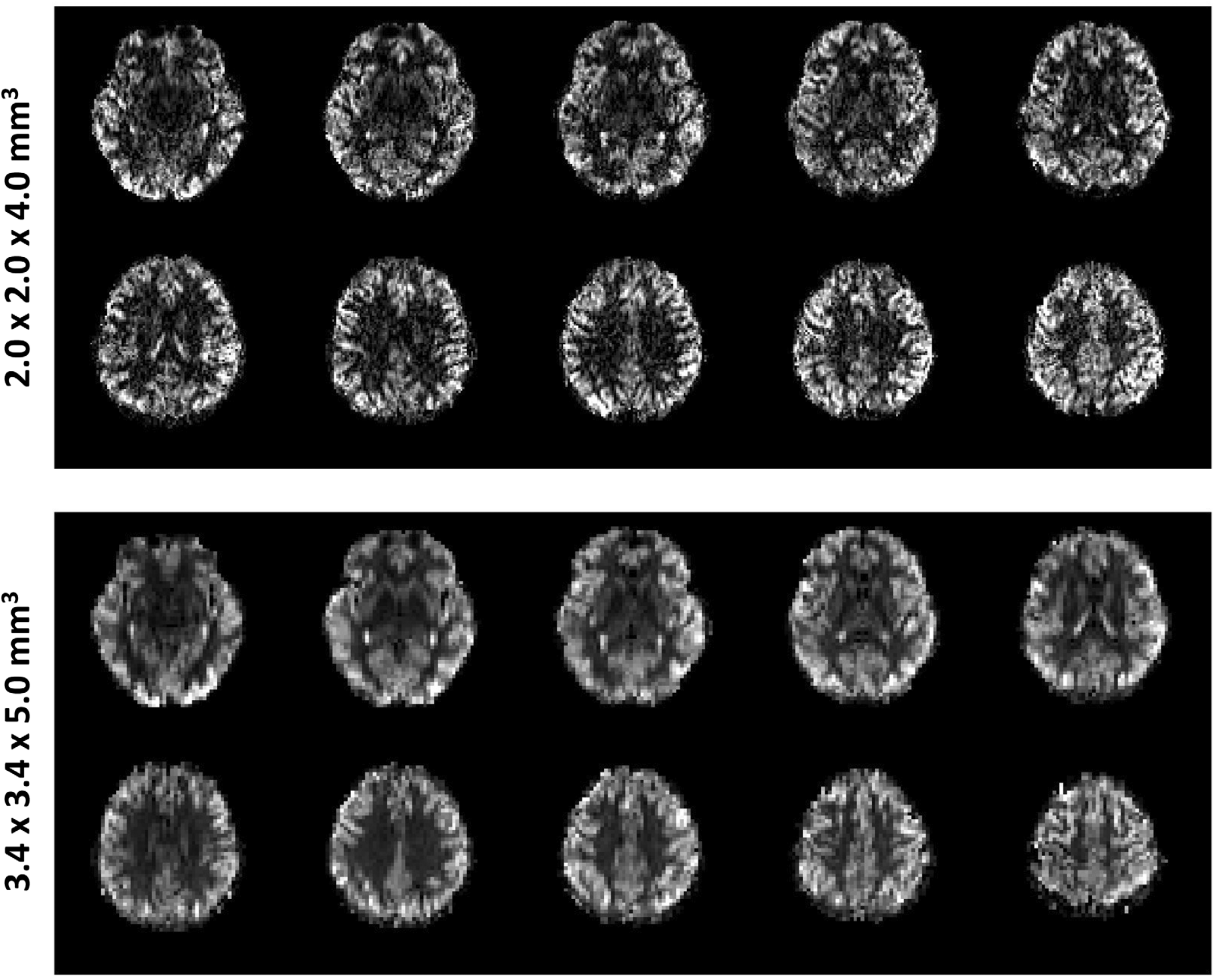
Demonstration of the capability of PCASL with 3D dynamic B_0_ shimming at 7 T to achieve high spatial resolution (2.0 × 2.0 × 4.0 mm^3^) relative CBF maps, compared with low spatial resolution (3.4 × 3.4 × 5.0 mm^3^) maps from a representative subject. The enhanced resolution enables improved visualization of fine‐scale CBF patterns. (The perfusion measurements are shown as relative values without units due to the absence of M_0_ calibration).

To demonstrate the benefits of the improved SNR from 7 T UHF‐PCASL, higher spatial resolution images were also acquired. Figure 8 demonstrates the ability of PCASL with the 3D dynamic B_0_ shimming method at 7 T to acquire high‐resolution perfusion maps. Data from a representative subject is shown. The high‐resolution maps provide finer anatomic details with a voxel size of 2.0 × 2.0 × 4 mm^3^ but at the cost of lower SNR compared to the low‐resolution reference maps with a voxel size of 3.4 × 3.4 × 5 mm^3^. This comparison highlights the potential of PCASL at 7 T for a more detailed investigation of CBF patterns in less than 5 min of scan time.

## 4 DISCUSSION

PCASL is recognized as the most preferred noninvasive perfusion technique among ASL variants at 3 T.^32^, ^33^, ^34^, ^35^ It employs a series of repeated low tip‐angle RF pulses for tagging, which are feasible to implement on modern clinical MRI scanners, unlike the continuous RF pulses required for CASL, while also significantly mitigating magnetization transfer effects.^4^, ^36^, ^37^ Compared to 3 T, PCASL at 7 T can provide an increase in SNR but in practice often exhibits a significant loss of perfusion signal due to off‐resonance issues within the labeling region positioned far from the isocenter. To address this challenge, our study introduces a novel dynamic B_0_ shimming method aimed at enhancing B_0_ field homogeneity, thereby improving the efficiency of arterial blood labeling.

Dynamic B_0_ shimming is also known as temporal control of the B_0_ shim fields or real‐time B_0_ shimming, which was initially developed to improve 2D multi‐slice MRI by customizing optimal shim settings for individual slices instead of settling for a global compromise.^38^, ^39^ This method was further used for addressing time‐varying B_0_ fluctuations caused by physiological processes such as respiration,^40^, ^41^, ^42^ where the shim fields need to be updated in sync with the respiratory cycle. However, the proposed dynamic B_0_ shimming in this study only requires switching the shim settings between the labeling and nonlabeling periods in the PCASL sequence. Because standard gradient coils in MRI systems serve dual roles in imaging and shimming, linear dynamic B_0_ shimming can be achieved by directly adjusting the gradient amplitudes, creating effects equivalent to modifying shim settings. However, this gradient‐based shimming approach is limited to correcting only the linear components of field inhomogeneity through gradient offset adjustments. After linear shimming, two types of field imperfections typically remain: a global frequency offset and nonlinear field inhomogeneities. To compensate for the global frequency offset, we added an additional phase term to the PCASL RF pulses, addressing the additional off‐resonance–induced phase buildup between successive pulses. Whereas we found this approach to be effective in the experiments performed for this study, it does not account for the actual labeling plane being shifted by ‐2πΔf /(γG_max_). For instance, with a 200 Hz offset (Δf) and a maximum gradient (G_max_) of 5.5 mT/m in the low SAR protocol, the labeling plane would shift by approximately 5 mm. An ideal approach to compensate for the global frequency offset involves adjusting the central frequency (also known as the zero‐order shimming parameter) during the labeling period by directly adding the Δf value to the central frequency. This targeted central frequency adjustment would eliminate the need to modify the RF pulse phase and avoid the undesired labeling plane shift.

In this study, the labeling plane was chosen in the neck, where the four main brain‐feeding arteries are located. Within this region, the arteries are relatively straight, and their geometry is simple. In such cases, the field inhomogeneity within the vessels can be well corrected by the linear terms using our targeted approach, with negligible contributions from nonlinear terms. However, PCASL and vessel‐encoded PCASL^43^ sometimes require placing the labeling plane above the circle of Willis, a region rich in intricate vascular structures.^44^ Unfortunately, this proximity to the air‐filled sinuses in the anterior of the brain significantly worsens field inhomogeneity. This, coupled with the need to label more vessels in this area, exacerbates the higher‐order field inhomogeneity within the target region, posing a substantial challenge for B_0_ shimming when only linear terms are used. Consequently, the proposed dynamic B_0_ shimming using the linear gradient would be unlikely to effectively address field inhomogeneity with significant nonlinear components. To address this, higher‐order spherical harmonic shimming coils may be employed.^45^ However, dynamic shimming with these coils requires careful monitoring and compensation for the eddy currents induced by shim coil switching, a process not typically performed for second‐order or higher‐order shim coils on modern clinical scanners.^46^ Meanwhile, an integrated RF/shim coil array, which shapes the B_0_ field by independently adjusting the direct current, can also be employed for dynamic B_0_ shimming in PCASL.^47^, ^48^ These arrays can be used separately or in combination with regular shimming coils.

It is valuable to compare our proposed dynamic B_0_ shimming method with others. Statistical analysis of the perfusion data in this study revealed that our 3D shimming method yielded a significantly higher perfusion signal than all the other evaluated methods. The 2D shimming method produces higher CBF than the OES‐based and global frequency correction methods, although this difference is not statistically significant. In terms of sequence implementation, our proposed method preserves the sequence structure, making it simpler to integrate. The principles of 2D dynamic B_0_ shimming are theoretically similar to existing 2D methods, such as the OES‐based^10^ method and OptPCASL,^14^ which apply gradient blips between PCASL RF pulses to correct the linear component of in‐plane field inhomogeneity. However, our dynamic B_0_ shimming also incorporates gradients along the z‐axis, allowing it to address through‐plane B_0_ variations, a feature unavailable in most other methods. Instead of relying on a simple shimming approach that extends the static shimming region to the edge of the labeling area, a different strategy could be applied to shim the labeling and imaging regions separately. This method represents another type of dynamic B_0_ shimming. However, it targets the entire labeling region, which includes static tissues, rather than focusing solely on the vessel voxels within, imposing greater constraints on the optimization process.

It is important to mention that B_1_^+^ inhomogeneity is another significant challenge for 7 T PCASL because it greatly affects labeling efficiency. B_1_^+^ varies with head size and positioning, exhibiting a rapid drop into the neck when using a head transmit coil at 7 T, along with different B_1_^+^ distributions at the feeding arteries for each subject.^49^ In our study, we attempted to mitigate the B_1_^+^ drop by increasing the RF flip angle by a certain factor. However, this approach did not address the in‐plane B_1_^+^ inhomogeneity across the labeled feeding arteries and may lead to variations in the labeling efficiency. Future studies could improve B_1_^+^ amplitude and its field homogeneity in the labeling plane by employing RF shimming with parallel transmission techniques or utilizing a dedicated labeling RF coil to enhance labeling efficiency at 7 T.^50^, ^51^, ^52^, ^53^ It is also worth noting that the GRE EPI readout used in this study may not be well optimized for 7 T imaging because distortions and signal dropouts can be observed in the EPI images. To improve image quality, dynamic B_0_ shimming with optimal shimming parameters for each slice can be employed during the readout period. Additionally, non‐EPI readout methods such as turbo‐FLASH^11^ can be utilized to improve image quality. Because the proposed dynamic B_0_ shimming method applies the gradient exclusively during the labeling period, it affects the labeling efficiency independently of the subsequent image readout. This generalized approach can be applied across any available readout methods and PCASL variants, including standard PCASL (both unbalanced and balanced) and vessel‐encoded PCASL. Whereas dynamic B_0_ shimming may have a smaller impact in PCASL settings that are less sensitive to B_0_ inhomogeneities, it still plays a significant role in reducing intersubject variability. Parameters that are less sensitive to B_0_ can only partially mitigate B_0_ inhomogeneities and may lose effectiveness when the field offset becomes large.^21^ Moreover, more B_0_‐robust settings often involve tradeoffs, such as increased SAR and reduced SNR efficiency. Notably, a pulsed gradient approach, which applies gradient blips between RF pulses, can serve as an alternative to constant gradients in dynamic B_0_ shimming to mitigate off‐resonant phase accrual. However, this method does not address the off‐resonance effects that occur during the PCASL RF pulses themselves.

The manual steps required in the current implementation of PCASL with dynamic B_0_ shimming limit its clinical feasibility. The workflow relies heavily on operator intervention, from aligning B_0_ shimming regions between field mapping and PCASL scans to selecting ROIs for feeding arteries and calculating shimming parameters offline. These manual processes not only increase the potential for errors but also extend scan duration, making the technique impractical for standard clinical protocols. To address these limitations, the field mapping sequence can be directly integrated into the PCASL scan as an automated pre‐scan step. Additionally, an automated process for selecting the labeling plane, defining vessel ROIs such as using artificial intelligence‐based methods, and calculating dynamic B_0_ shimming automatically should be developed and deployed directly on the scanner console. These integrated features would substantially streamline the PCASL workflow with dynamic B_0_ shimming, enhancing operational efficiency and user accessibility, thereby facilitating broader clinical adoption. In this study, we evaluated our method on healthy young volunteers. However, in clinical scenarios involving pathology or aging populations, vessel narrowing may reduce the available voxel count within the vessels. This reduction can make the calculation of the linear shimming term more susceptible to noise and flow‐related artifacts, potentially compromising accuracy. To address challenges associated with limited vessel voxels, we propose expanding the ROIs to encompass both the vessel and surrounding static tissue, leveraging the inherently smooth characteristics of field maps. Future studies will systematically evaluate the efficacy of this approach.

## 5 CONCLUSION

Our study presents a novel dynamic B_0_ shimming approach that significantly enhances PCASL performance at 7 T. This method effectively addresses both in‐plane and through‐plane B_0_ variations within the labeling region. Importantly, our approach specifically targets B_0_ homogeneity within the vessels of interest within the labeling region, rather than the entire labeling area, allowing for more precise optimization compared to conventional whole‐region dynamic shimming approaches. Overall, this rapid and focused B_0_ shimming strategy significantly improves the robustness and effectiveness of PCASL, unlocking the inherent advantages of 7 T ASL high sensitivity and spatial resolution for enhanced perfusion imaging.

## Data Availability

The code for the dynamic shimming calculations can be found at https://github.com/yangji6/Dynamic‐B0‐Shimming‐for‐ASL. Data underlying the plots in this paper can be found at https://doi.org/10.5281/zenodo.13879457.

## References

[1] Detre JA , Leigh JS , Williams DS , Koretsky AP . Perfusion imaging. Magn Reson Med. 1992;23:37‐45. 1734182 10.1002/mrm.1910230106

[2] Williams DS , Detre JA , Leigh JS , Koretsky AP . Magnetic resonance imaging of perfusion using spin inversion of arterial water. Proc Natl Acad Sci U S A. 1992;89:212‐216. 1729691 10.1073/pnas.89.1.212 PMC48206

[3] Dai W , Garcia D , De Bazelaire C , Alsop DC . Continuous flow‐driven inversion for arterial spin labeling using pulsed radio frequency and gradient fields. Magn Reson Med. 2008;60:1488‐1497. 19025913 10.1002/mrm.21790 PMC2750002

[4] Wong EC . An introduction to ASL labeling techniques. J Magn Reson Imaging. 2014;40:1‐10. 24424918 10.1002/jmri.24565

[5] Teeuwisse WM , Webb AG , van Osch MJ . Arterial spin labeling at ultra‐high field: all that glitters is not gold. Int J Imaging Syst Technol. 2010;20:62‐70.

[6] Le Ster C , Grant A , Van de Moortele PF , et al. Magnetic field strength dependent SNR gain at the center of a spherical phantom and up to 11. 7T. Magn Reson Med. 2022;88:2131‐2138. 35849739 10.1002/mrm.29391 PMC9420790

[7] Li W , Grgac K , Huang A , Yadav N , Qin Q , van Zijl PC . Quantitative theory for the longitudinal relaxation time of blood water. Magn Reson Med. 2016;76:270‐281. 26285144 10.1002/mrm.25875 PMC4758918

[8] Wang K , Shao X , Yan L , Ma SJ , Jin J , Wang DJ . Optimization of adiabatic pulses for pulsed arterial spin labeling at 7 Tesla: comparison with pseudo‐continuous arterial spin labeling. Magn Reson Med. 2021;85:3227‐3240. 33427349 10.1002/mrm.28661 PMC8351166

[9] Jahanian H , Noll DC , Hernandez‐Garcia L . B0 field inhomogeneity considerations in pseudo‐continuous arterial spin labeling (pCASL): effects on tagging efficiency and correction strategy. NMR Biomed. 2011;24:1202‐1209. 21387447 10.1002/nbm.1675

[10] Berry ES , Jezzard P , Okell TW . Off‐resonance correction for pseudo‐continuous arterial spin labeling using the optimized encoding scheme. Neuroimage. 2019;199:304‐312. 31158481 10.1016/j.neuroimage.2019.05.083 PMC6892252

[11] Zhao C , Shao X , Shou Q , et al. Whole‐cerebrum distortion‐free three‐dimensional pseudo‐continuous arterial spin labeling at 7T. Neuroimage. 2023;277:120251. 37364741 10.1016/j.neuroimage.2023.120251 PMC10528743

[12] Zhao L , Vidorreta M , Soman S , Detre JA , Alsop DC . Improving the robustness of pseudo‐continuous arterial spin labeling to off‐resonance and pulsatile flow velocity. Magn Reson Med. 2017;78:1342‐1351. 27774656 10.1002/mrm.26513 PMC5848499

[13] Jung Y , Wong EC , Liu TT . Multiphase pseudocontinuous arterial spin labeling (MP‐PCASL) for robust quantification of cerebral blood flow. Magn Reson Med. 2010;64:799‐810. 20578056 10.1002/mrm.22465

[14] Shin DD , Liu TT , Wong EC , Shankaranarayanan A , Jung Y . Pseudocontinuous arterial spin labeling with optimized tagging efficiency. Magn Reson Med. 2012;68:1135‐1144. 22234782 10.1002/mrm.24113 PMC3345172

[15] Luh WM , Talagala SL , Li TQ , Bandettini PA . Pseudo‐continuous arterial spin labeling at 7 T for human brain: estimation and correction for off‐resonance effects using a prescan. Magn Reson Med. 2013;69:402‐410. 22488568 10.1002/mrm.24266 PMC3402610

[16] Berry ES , Jezzard P , Okell TW . An optimized encoding scheme for planning vessel‐encoded pseudocontinuous arterial spin labeling. Magn Reson Med. 2015;74:1248‐1256. 25351616 10.1002/mrm.25508

[17] Ji Y , Li H , Woods J , Okell T . Dynamic B0 field shimming for improving pseudo‐continuous arterial spin labeling at 7 Tesla. In Proceedings of the 32nd Annual Meeting of ISMRM, Singapore, 2024. p. 2019.

[18] Hirschler L , Debacker CS , Voiron J , Köhler S , Warnking JM , Barbier EL . Interpulse phase corrections for unbalanced pseudo‐continuous arterial spin labeling at high magnetic field. Magn Reson Med. 2018;79:1314‐1324. 28585234 10.1002/mrm.26767

[19] Okell TW , Chappell MA , Kelly ME , Jezzard P . Cerebral blood flow quantification using vessel‐encoded arterial spin labeling. J Cerebr Blood Flow Metab. 2013;33:1716‐1724. 10.1038/jcbfm.2013.129 PMC3824178 23921895

[20] Ehses P , Brenner D , Stirnberg R , Pracht ED , Stöcker T . Whole‐brain B1‐mapping using three‐dimensional DREAM. Magn Reson Med. 2019;82:924‐934. 31038244 10.1002/mrm.27773

[21] Saïb G , Koretsky AP , Talagala SL . Optimization of pseudo‐continuous arterial spin labeling using off‐resonance compensation strategies at 7T. Magn Reson Med. 2022;87:1720‐1730. 34775619 10.1002/mrm.29070 PMC8810716

[22] Woods J , Chiew M , Okell T . Minimizing SAR for SNR‐efficient pseudo‐continuous arterial spin labeling at 7T. In Proceedings of the 31st Annual Meeting of ISMRM, Toronto, Ontario, Canada, 2023. p. 371.

[23] Meixner CR , Eisen CK , Schmitter S , et al. Hybrid‐shimming and gradient adaptions for improved pseudo‐continuous arterial spin labeling at 7 Tesla. Magn Reson Med. 2022;87:207‐219. 34411335 10.1002/mrm.28982

[24] Boland M , Stirnberg R , Pracht ED , Stöcker T . Robust and SAR‐efficient whole‐brain pseudo‐continuous ASL at 7T. In Proceedings of the 27th Annual Meeting of ISMRM, Montréal, Québec, Canada, 2019. p. 4963.

[25] Günther M , Oshio K , Feinberg DA . Single‐shot 3D imaging techniques improve arterial spin labeling perfusion measurements. Magn Reson Med. 2005;54:491‐498. 16032686 10.1002/mrm.20580

[26] Griswold MA , Jakob PM , Heidemann RM , et al. Generalized autocalibrating partially parallel acquisitions (GRAPPA). Magn Reson Med. 2002;47:1202‐1210. 12111967 10.1002/mrm.10171

[27] Jenkinson M , Beckmann CF , Behrens TE , Woolrich MW , Smith SM . FSL . Neuroimage. 2012;62:782‐790. 21979382 10.1016/j.neuroimage.2011.09.015

[28] Jenkinson M , Bannister P , Brady M , Smith S . Improved optimization for the robust and accurate linear registration and motion correction of brain images. Neuroimage. 2002;17:825‐841. 12377157 10.1016/s1053-8119(02)91132-8

[29] Chappell MA , Groves AR , Whitcher B , Woolrich MW . Variational Bayesian inference for a nonlinear forward model. IEEE Trans Signal Process. 2008;57:223‐236.

[30] Chappell MA , Kirk TF , Craig MS , et al. BASIL: a toolbox for perfusion quantification using arterial spin labelling. Imaging Neurosci. 2023;1:1‐16. 10.1162/imag_a_00041 PMC12007520 40799705

[31] Okell TW . Simulator for MRI pulse sequences: Initial release (v1.0.0). Zenodo 2023. doi:10.5281/zenodo.7952965

[32] Woods JG , Achten E , Asllani I , et al. Recommendations for quantitative cerebral perfusion MRI using multi‐timepoint arterial spin labeling: acquisition, quantification, and clinical applications. Magn Reson Med. 2024;92:469‐495. 38594906 10.1002/mrm.30091 PMC11142882

[33] Suzuki Y , Clement P , Dai W , et al. ASL lexicon and reporting recommendations: a consensus report from the ISMRM Open Science initiative for perfusion imaging (OSIPI). Magn Reson Med. 2024;91:1743‐1760. 37876299 10.1002/mrm.29815 PMC10950547

[34] Lindner T , Bolar DS , Achten E , et al. Current state and guidance on arterial spin labeling perfusion MRI in clinical neuroimaging. Magn Reson Med. 2023;89:2024‐2047. 36695294 10.1002/mrm.29572 PMC10914350

[35] Alsop DC , Detre JA , Golay X , et al. Recommended implementation of arterial spin‐labeled perfusion MRI for clinical applications: a consensus of the ISMRM perfusion study group and the European consortium for ASL in dementia. Magn Reson Med. 2015;73:102‐116. 24715426 10.1002/mrm.25197 PMC4190138

[36] Ji Y , Lu D , Jiang Y , Wang X , Meng Y , Sun PZ . Development of fast multi‐slice apparent T1 mapping for improved arterial spin labeling MRI measurement of cerebral blood flow. Magn Reson Med. 2021;85:1571‐1580. 32970848 10.1002/mrm.28510 PMC7718392

[37] Jezzard P , Chappell MA , Okell TW . Arterial spin labeling for the measurement of cerebral perfusion and angiography. J Cerebr Blood Flow Metab. 2018;38:603‐626. 10.1177/0271678X17743240 PMC5888859 29168667

[38] Blamire AM , Rothman DL , Nixon T . Dynamic shim updating: a new approach towards optimized whole brain shimming. Magn Reson Med. 1996;36:159‐165. 8795035 10.1002/mrm.1910360125

[39] Morrell G , Spielman D . Dynamic shimming for multi‐slice magnetic resonance imaging. Magn Reson Med. 1997;38:477‐483. 9339449 10.1002/mrm.1910380316

[40] Stockmann JP , Wald LL . In vivo B0 field shimming methods for MRI at 7 T. Neuroimage. 2018;168:71‐87. 28602943 10.1016/j.neuroimage.2017.06.013 PMC5760477

[41] Sengupta S , Welch EB , Zhao Y , et al. Dynamic B0 shimming at 7 T. Magn Reson Imaging. 2011;29:483‐496. 21398062 10.1016/j.mri.2011.01.002 PMC3078963

[42] D'Astous A , Cereza G , Papp D , et al. Shimming toolbox: an open‐source software toolbox for B0 and B1 shimming in MRI. Magn Reson Med. 2023;89:1401‐1417. 36441743 10.1002/mrm.29528 PMC9910837

[43] Wong EC . Vessel‐encoded arterial spin‐labeling using pseudocontinuous tagging. Magn Reson Med. 2007;58:1086‐1091. 17969084 10.1002/mrm.21293

[44] Okell TW , Garcia M , Chappell MA , Byrne JV , Jezzard P . Visualizing artery‐specific blood flow patterns above the circle of Willis with vessel‐encoded arterial spin labeling. Magn Reson Med. 2019;81:1595‐1604. 30357925 10.1002/mrm.27507 PMC6492185

[45] Hetherington HP , Moon CH , Schwerter M , Shah NJ , Pan JW . Dynamic B0 shimming for multiband imaging using high order spherical harmonic shims. Magn Reson Med. 2021;85:531‐543. 32857424 10.1002/mrm.28438 PMC8098050

[46] Koch KM , McIntyre S , Nixon TW , Rothman DL , de Graaf RA . Dynamic shim updating on the human brain. J Magn Reson. 2006;180:286‐296. 16574443 10.1016/j.jmr.2006.03.007

[47] Han H , Song AW , Truong TK . Integrated parallel reception, excitation, and shimming (iPRES). Magn Reson Med. 2013;70:241‐247. 23629974 10.1002/mrm.24766 PMC3800110

[48] Stockmann JP , Witzel T , Keil B , et al. A 32‐channel combined RF and B0 shim array for 3T brain imaging. Magn Reson Med. 2016;75:441‐451. 25689977 10.1002/mrm.25587 PMC4771493

[49] de Buck MH , Kent JL , Jezzard P , Hess AT . Head‐and‐neck multichannel B1+ mapping and RF shimming of the carotid arteries using a 7T parallel‐transmit head coil. Magn Reson Med. 2024;91:190‐204. 37794847 10.1002/mrm.29845 PMC10962593

[50] Wang K , Ma SJ , Shao X , et al. Optimization of pseudo‐continuous arterial spin labeling at 7T with parallel transmission B1 shimming. Magn Reson Med. 2022;87:249‐262. 34427341 10.1002/mrm.28988 PMC8616784

[51] Tong Y , Jezzard P , Okell TW , Clarke WT . Improving PCASL at ultra‐high field using a VERSE‐guided parallel transmission strategy. Magn Reson Med. 2020;84:777‐786. 31971634 10.1002/mrm.28173 PMC7216913

[52] Mora Álvarez MG , Stobbe RW , Beaulieu C . High resolution continuous arterial spin labeling of human cerebral perfusion using a separate neck tagging RF coil. PLoS One. 2019;14:e0215998. 31022257 10.1371/journal.pone.0215998 PMC6483248

[53] Hirschler L , Collomb N , Voiron J , Köhler S , Barbier EL , Warnking JM . SAR comparison between CASL and pCASL at high magnetic field and evaluation of the benefit of a dedicated labeling coil. Magn Reson Med. 2020;83:254‐261. 31429990 10.1002/mrm.27931

